# A case of middle mediastinal cavernous hemangioma

**DOI:** 10.1111/1759-7714.13301

**Published:** 2020-01-16

**Authors:** Xianqi Cai, Chunquan Liu, Yong Cui

**Affiliations:** ^1^ Department of Thoracic Surgery Beijing Friendship Hospital, Capital Medical University Beijing China

**Keywords:** Cavernous hemangioma, middle mediastinal, surgical resection

## Abstract

While cavernous hemangioma are frequently observed in various tissues and locations in the body, mediastinal cavernous hemangiomas (CHMs) are rare, particularly in the middle mediastinum. Here, we report a case of a middle CHM which was diagnosed and treated in our hospital. A male patient age 57 years was admitted with a mediastinal circular low‐density lesion. Preoperative examination was performed with a subsequent diagnosis of a mediastinal lesion. The lesion was resected and post‐operative histopathology suggested that it was a cavernous hemangioma. Post‐operative recovery was uneventful, and a follow‐up examination nearly one year later showed that the patient had no recurrence.

## Introduction

A mediastinal cavernous hemangioma (CHM) is a rare benign tumor caused by congenital vascular dysplasia, which accounts for less than 0.5% of mediastinal tumors. The disease mostly occurs in young adults, and the gender difference is not large. More than 50% of CHM are located in the anterior mediastinum, followed by the posterior mediastinum, while the middle mediastinum is the least common location. Here, we report the case of a 57‐year‐old male diagnosed with a middle mediastinal cavernous hemangioma at our hospital.

## Case report

**Figure 1:**
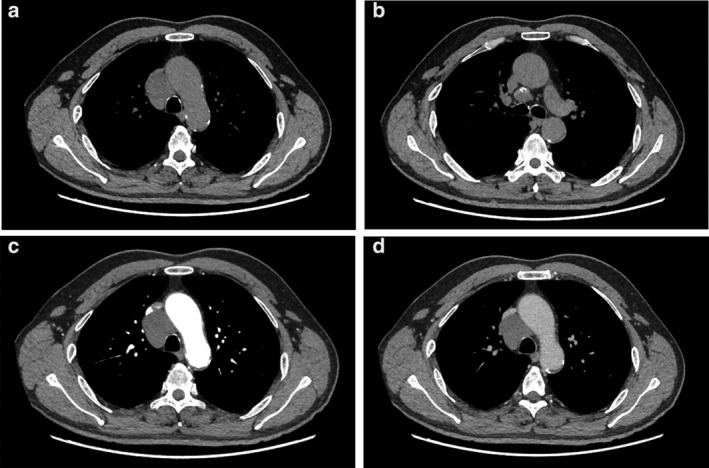
(**a**) An irregular slightly low‐density shadow appeared in the anterior trachea and posterior superior vena cava of the mediastinum on chest enhanced computed tomography (CT). The lesion boundary was clear. (**b**) A small calcification and solid component were visualized at the lower edge of the lesion. The lesion measured 5.3 cm × 4.4 cm × 4.8 cm (upper and lower × front and rear × left and right), and the scan CT value was approximately 25 HU, enhanced CT value approximately 36 HU. (**c**,**d**) A small piece of solid component was clearly enhanced.

**Figure 2:**
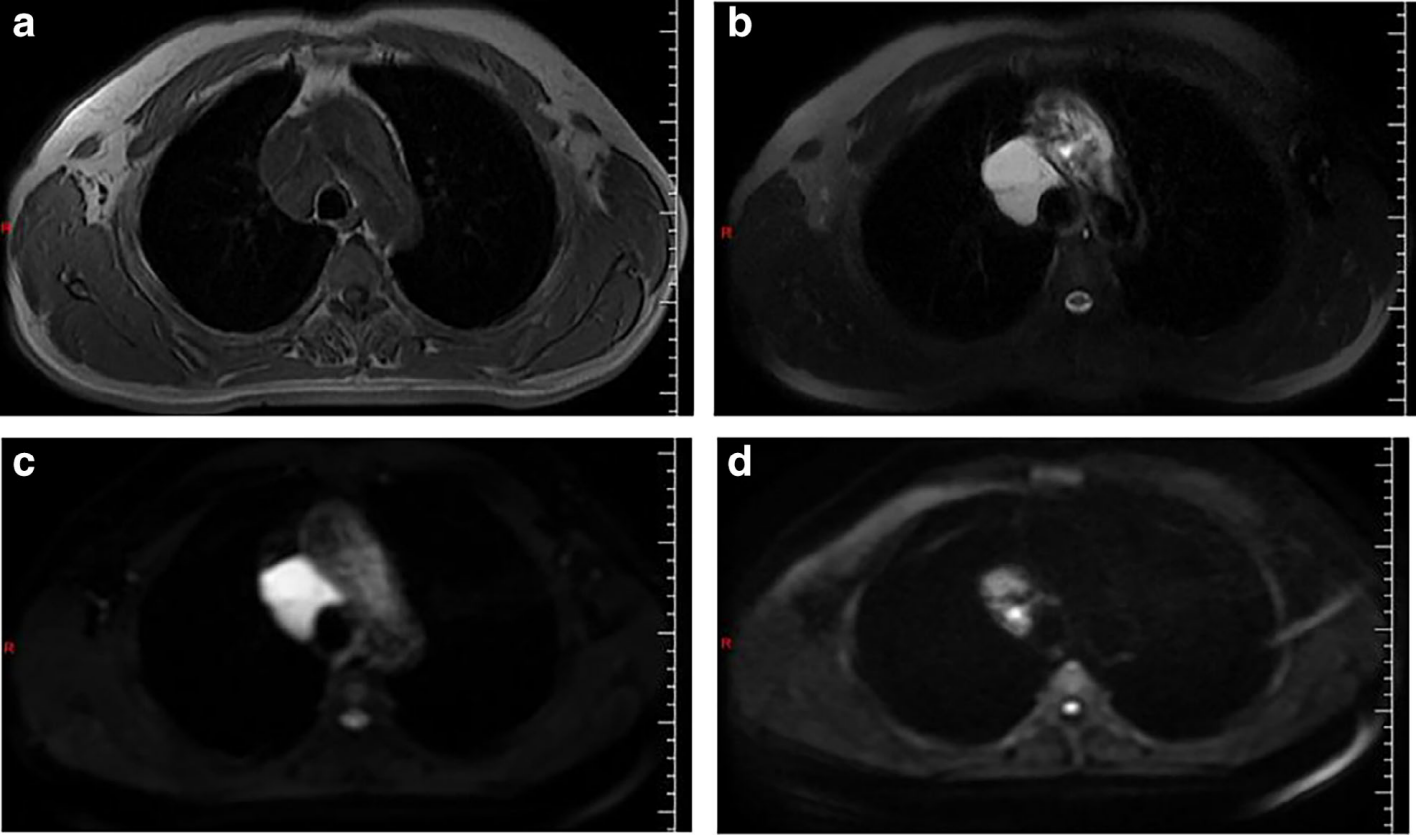
Irregular abnormal signals were seen in the vena cava‐anterior trachea space on chest MRI (**a**) with uniformly low signals on T1WI (**b**) and significantly high signals on T2WI. There was no obvious diffusion limitation of lesions on different b‐value DWI. (**c**,**d**) The lesion considers vascular lesion.

**Figure 3:**
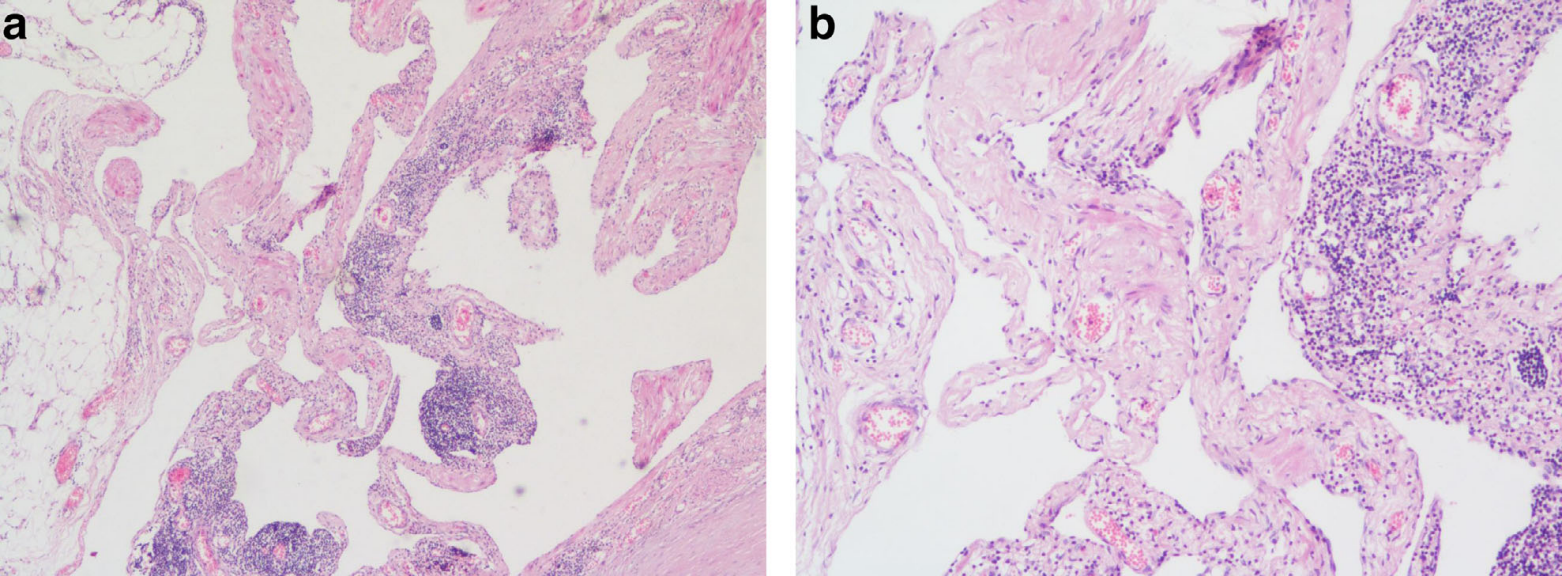
Postoperative pathology. (**a**) Hematoxylin and eosin stain (magnification ×40), (**b**) hematoxylin and eosin stain (magnification ×100): Mediastinal mass: fibrous adipose tissue (3 × 2 × 0.8 cm) with dilated vascular lumen, thickened wall, smooth muscle hyperplasia, lumen expansion with peripheral lymphoid hyperplasia. It was considered to be a cavernous hemangioma, and also showed reactive lymphoid hyperplasia.

A 57‐year‐old male patient was admitted to hospital following a routine medical examination with a history of a mediastinal lesion having been located on examination at his local hospital one week previously. Chest computed tomography (CT) scan had confirmed a circular low‐density lesion in the mediastinum. The patient presented with no evidence of fever, or other symptoms such as chest pain, dyspnea, or dysphagia. In order to determine a definite diagnosis and treatment, the patient subsequently visited our hospital. On arrival, the patient confirmed that there was no history of chest lesions in the family. Chest auscultation and percussion were essentially negative. Cardiopulmonary function test was generally normal, and tumor index NSE (neuron specific enolase) was 24.11 ng/mL (normal reference range 0.00–18.00 ng/mL). Chest‐enhanced CT (Fig 1) displayed an irregular slightly low‐density shadow in the anterior trachea and posterior superior vena cava of the mediastinum. The boundary of the lesion was clear, although a small calcification and solid component were visible at the lower edge of the lesion. The scan CT value was approximately 25 HU, enhanced CT value 36 HU, and a small piece of solid component below was clearly enhanced. Diagnosis revealed a slightly lower density lesion of the anterior tracheal and posterior vena cava in the mediastinum, the nature of which was uncertain. Chest MRI (Fig 2) of the patient indicated that irregular abnormal signals were seen in the vena cava‐anterior trachea space, with uniformly low signals on T1WI and significantly high signals on T2WI. There was no obvious diffusion limitation of lesions on different b‐value DWI, the lesion considers vascular lesion. There was a clear indication that the patient be recommended for surgery, and thoracoscopic resection of the mediastinal lesion was recommended as the treatment plan. Following usual preoperative procedures, the patient was anaesthetized and placed on his left side on the operating table .A 4 cm incision was made into the chest, entering via the fourth intercostal space and a thoracoscopy was performed. On visualization, there was no adhesion in the chest, but there was a visible lesion in the middle mediastinum. An ultrasonic knife was used to anatomize the lesion from the superior vena cava and the azygous arch, with careful dissection and separation of the lesion under thoracoscopy. The size of the lesion was approximately 6 cm × 5 cm × 3 cm, with visible localized calcification. The lesion was removed for routine pathology. Postoperative pathology (Fig 3) confirmed that it was a mediastinal lesion with fibrous adipose tissue (3 × 2 × 0.8 cm), dilated vascular lumen diagnosis, and the lesion was considered to be a cavernous hemangioma. The patient was successfully discharged from hospital on the fifth day after surgery, with no evidence of recurrence after one year of follow‐up.

## Discussion

A mediastinal cavernous hemangioma (CHM) is a rare benign tumor caused by congenital vascular dysplasia, and accounts for less than 0.5% of mediastinal tumors. Histopathological features of CHM include a composition of cystic masses made up of dilated cavernous sinusoids of varying sizes, with the inner wall lined with vascular endothelium. More than 50% of lesions are located in the anterior mediastinum, followed by the posterior mediastinum, with the middle mediastinum being the least common location.^1^ In the case reported here, the patient's lesion was located in the middle mediastinum, anterior trachea and posterior superior vena cava, and the history for this presentation is extremely rare. The Pubmed database was searched, and a total of six cases of middle mediastinal cavernous hemangiomas were retrieved.

The clinical symptoms of CHM are related to their size, location and nature with usually no obvious symptoms in the early stages. Following enlargement of the tumor, certain symptoms of compression often appear. Zeyaian *et al*.^2^ reported symptoms of spinal cord or nerve root compression in patients with posterior mediastinal cavernous hemangioma, including local pain, radiculopathy, and slow progressive or acute spinal cord compression. Deepak *et al*.^3^ reported a fever and cough in a four‐year‐old boy whose chest CT showed an anterior mediastinal source, accounting for approximately three quarters of the right thoracic cavity, and was confirmed as a mediastinal hemangioma on postoperative pathology. Although the lesion was located in the anterior trachea and posterior superior vena cava, there was no compression on surrounding tissue, so there were no relevant compression symptoms.

Kim *et al*.^4^ and He *et al*.^5^ reported a cavernous hemangioma located in the mediastinum and heart, which may cause chest embolism due to cardiac tamponade and pericardium. CHM has a multicenter morbidity tendency, often combined with hemangioma lesions in other parts of the skin, liver, spleen, and kidney. However, most patients have no obvious symptoms. Mediastinal space‐occupying lesions in the majority of patients are discovered on chest X‐ray, CT or magnetic resonance imaging. The nature of these lesions is not entirely clear. Preoperative diagnosis of mediastinal cavernous hemangioma is particularly difficult. General chest X‐ray diagnosis of mediastinal cavernous hemangioma is not very useful. Li *et al*.^6^ reported the value of multiphase enhanced CT being used to diagnose a hemangioma, where the tumor showed peripheral nodular enhancement on early phase images and progressive centripetal fill‐in on delayed phase images. A phlebolith is a characteristic manifestation of CHM which usually present as a small nodular or needle‐like shape. It is formed by thrombosis of calcification in the sinusoidal cavity and has certain characteristics. The probability of a phlebolith appearing is approximately 20% and they usually present as spotted high density shadows. In this patient's lesion, there was a calcification visible on the CT and a small patchy enhancement on the edge of the arterial phase, suggesting a mediastinal hemangioma lesion. Transesophageal ultrasonography, endobronchial ultrasonography and angiography^7^ are also a means of confirming mediastinal disease and diagnosing mediastinal lesions, but due to their invasive nature, they are not widely used in clinical practice.

In the diagnosis of CHM, there are common differential diagnoses: (i) Mediastinal lymphoma^8^: this is a malignant tumor originating from lymphoid tissue, and the common clinical manifestations are cough, hypothermia, fatigue, etc often accompanied by multiple superficial lymph node pain and enlargement, mostly in the incidence of young and middle‐aged. Chest CT features are bilateral paratracheal, posterior sternal and hilar lymph node enlargement. CHM does not usually have the lymph nodes mentioned which can be identified according to the symptoms and CT characteristics of the chest. (ii) Castleman disease^9^: this is a rare lymphoid tissue proliferative disease, with a tumor‐like lesion, also known as vascular follicular lymphoid tissue hyperplasia, mainly involving the chest, lung and mediastinum, abdominal cavity etc. Some patients may have compression symptoms caused by lymphadenopathy due to the rich blood supply of giant lymph node hyperplasia. An enhanced shadow may be visualized on an arterial phase enhanced CT, but identification of CHM and CD mainly rely on postoperative pathological diagnosis.

Although CHM is benign in histological structure, some lesions may show invasive growth, which may cause complications such as hemorrhage or asphyxia.^10^ Complete surgical resection is a radical treatment. Most CHM have a complete capsule and are easy to separate.^11^ Thoracoscopy can be used to remove lesions; some tumors are huge, or are closely adhererent to large blood vessels, pericardium, etc with an abundant blood supply and they should be carefully dissected during surgery, under thoracoscopy. When the lesion is difficult to remove, the chest should be opened in time to ensure the safety of the operation. The patient in our study underwent thoracoscopic resection of the lesion. Interventional embolization is a method for the treatment of hemangioma, and even though microinvasive surgery is safe and effective, in the case of CHM, there have been few clinical applications because it is difficult to obtain a pathological diagnosis before surgery. Complete resection of CHM has a good prognosis and generally it is not common for there to be a recurrence.^12^ This patient had no recurrence after one year of follow‐up. Although the disease is benign and rarely malignant, follow‐up is needed within one year to observe recurrence.

## Disclosure

The authors declare that there are no conflicts of interest.
